# The mysterious sex chromosomes of haploid plants

**DOI:** 10.1038/s41437-022-00524-2

**Published:** 2022-04-07

**Authors:** Deborah Charlesworth

**Affiliations:** Institute of Evolutionary Biology, School of Biological Sciences, Ashworth Lab, University of Edinburgh, Edinburgh, UK

**Keywords:** DNA sequencing, Evolutionary genetics

The sex chromosomes of haploid plants, including mosses and liverworts (members of the bryophytes) have been much less studied than those of diploid plants. However, it has long been known that having separate sexed individuals is much commoner in bryophytes (Perley and Jesson 2015), than in diploid plants (in which the proportion of dioecious species has consistently been estimated at around 5% (see Charlesworth 1985, Renner 2014, Yampolsky and Yampolsky 1922). Some plants of both types may not have genetic sex-determination (Pannell 1997, Tanurdzic and Banks 2004, Zimmerman 1991), but early cytogenetic studies in bryophytes observed heteromorphic sex chromosomes (Allen 1917; 1919), with the karyotypes of male and female gametophytes differing clearly. Unbiased estimates are not yet available, and species numbers studied are small, but the data (reviewed by Allen (1945) and Renner et al. (2017)) suggest that around half of separate sexed bryophyte species have visibly different sex chromosomes, at least as many as in dioecious diploid angiosperms (Ming et al. 2011, Westergaard 1958). Overall, therefore, many more bryophyte than angiosperm species are available for studying sex chromosomes.

**Fig. 1:**
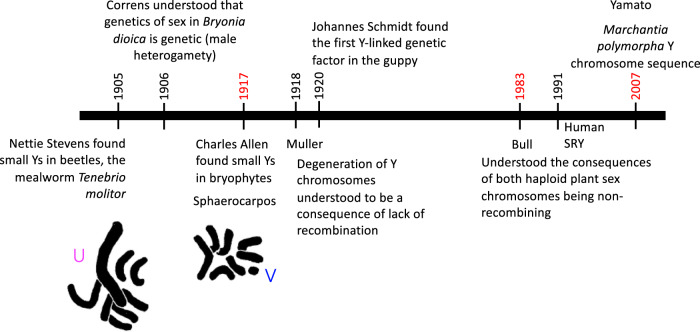
Sex chromosome discoveries. The karyotype of the bryophyte *Sphaerocarpos* is from Allen (1917).

Bryophyte sex chromosomes have, however, been surprisingly neglected (Fig. 1), especially as the dominance of the haploid life cycle stage, the gametophyte stage, predicts interesting differences from the sex chromosomes of diploid plants or animals (Table 1 below). Moreover the free-living gametophytes are large, and can be collected and their genome sequences provide phased data, allowing the two sex chromosome sequences to be assembled separately, which is difficult in diploids where the Y chromosome has to be assembled in the XY males (or in ZW females in species with female heterogamety).

**Table 1:** Predictions about gene losses from U and V chromosomes.

Gene expression	Loss from U (female-determining)	Loss from V (male-determining)
Male gametophyte-specific	Yes	No
Female gametophyte-specific	No	Yes
Gametophyte vegetative stage	Unlikely, but function may deteriorate	
Sporophyte specific: such a gene can be lost from the U or the V, but not both	Yes	Yes

Most genes should be retained on both the U and V.

**Fig. 2:**
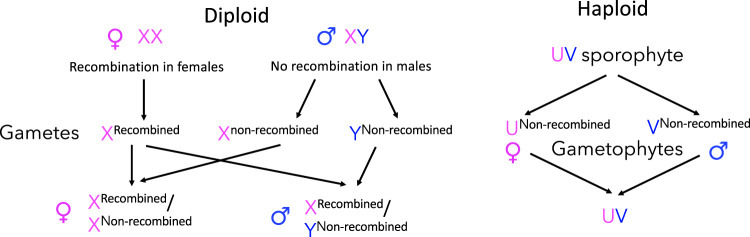
Genetics of completely sex-linked regions. Genetics in diploids (left), with X chromosome recombination in females each generation, but lack of recombination in the fully Y-linked region, and in haploid plants (right), showing that, like completely Y-linked regions, both fully U- and V-linked regions cannot become homozygous and recombine.

Bryophyte sex chromosome sequences offer the opportunity to test the differences predicted between haploid and diploid systems (Bull 1978). Bull pointed out that, in the haploid plant life cycle, the diploid zygote ‘is produced by the union of a gamete from a female and one from a male and is therefore always heterozygous, XY. The diploid merely produces haploid spores’. Consequently, if a non-recombining, completely sex-linked region exists, both the male- and female-determining versions are non-recombining, unlike in diploid flowering plants (or animals with XY systems), which have fully Y-linked regions, while the X-linked region crosses over in females. To emphasise this difference, the sex chromosomes in female and male gametophyte are no longer called X and Y, respectively, but are named U and V (Fig. 2).

The recombination asymmetry between the sexes in diploids can explain why only Y chromosomes undergo genetic degeneration (Muller 1914). Since Y-linked mutations cannot cross over onto the X, they will always be heterozygous (unless the same mutation happens to arise on the X, which is highly unlikely). Deleterious Y-linked mutations can persist (or even spread through the Y population) because selection against them is weak, since only their heterozygous effects are expressed. The Y population is isolated from the (larger) X population, allowing greater effects of genetic drift on the Y than other chromosomes, which also contributes to processes causing genetic degeneration, as reviewed by Bachtrog (2008).

In haploid plants, Muller’s theory applies equally to both sex-linked regions in the diploid stage, and mutations can persist on either chromosome, both of which might be expected to degenerate to about the same extent, some genes degenerating in function, or even being lost, from one or the other region. However, selection in the haploid stage, where mutations are not ‘sheltered’ by being heterozygous with functional alleles, might be predicted to limit degeneration involving mutations with strongly deleterious effects on fitness. Bull therefore predicted that, although both sex chromosomes should degenerate to similar extents, degeneration should be minor on both, and neither should involve losses of many genes. He therefore also predicted that, if the sex-linked regions differ in size, this should mainly reflect additions, perhaps adding genes beneficial to one sex of gametophyte. However, the diminutive sex-specific chromosomes detected cytogenetically (Allen 1917; 1919) suggest major losses of genes. These different predictions (Table 1) can now be evaluated by genome sequence analyses together with transcriptome results to show which genes are expressed specifically in one sex of gametophyte, or specifically in sporophytes, and are most likely to be lost from either the U- or the V-linked region.

Sequencing studies are now starting to be published. Recently, results have appeared for two mosses from the group Dicranidae, *Syntrichia caninervis* (Silva et al. 2021) and *Ceratodon purpureus* (Carey et al. 2021). It turns out to be harder than expected to understand them, even though both species’ genomes are only around 300 Mb in total, and their repetitive sequence densities are not high enough, overall, to cause major assembly problems. Although neither species has visible sex chromosome heteromorphism (they are ‘homomorphic’), the sex chromosomes are highly distinctive and differ from the autosomes; in both species, they are the largest chromosomes in both male and female gametophytes. The *C. purpureus* study focuses on the sex chromosomes (Carey et al. 2021), whose U and V assembly sizes are 110.5 and 112.2 Mb, respectively. Both have much higher repetitive content (around 80%) than the autosomes (slightly below 50%), and consequently lower gene densities. Their low gene densities reflect the repeat accumulation expected in non-recombining regions (Charlesworth et al. 1994), not gene losses, and, as predicted, both sex chromosomes in these moss species share this characteristic. Repeat accumulation may contribute to chromosome rearrangements by allowing recombination events between sequences in different genome locations, particularly as rearrangements are not disadvantageous in the absence of crossing over (Charlesworth et al. 1994). The gene orders are indeed very different in the U and V assemblies.

Both sex chromosomes nevertheless carry large numbers of genes (contributing transcript numbers representing ~12% of the total gene content), apparently supporting the prediction that degeneration should be minor. However, a whole genome duplication event (WGD) early in moss evolutionary history (Gao et al. 2020) complicates the situation. This doubled the chromosome number after the split from liverworts. In moss species, each chromosome should therefore be represented as two homeologues, though, if the event occurred long enough ago, individual genes may no longer be present as duplicates (as ‘diploidisation’ processes generally return such ‘paleopolyploids’ to ‘genetic diploidy’, for example in teleost fish, see Braasch et al. 2015, Jaillon et al. 2004).

Unexpectedly, however, *C. purpureus* has only one of each type of sex chromosome. Perhaps the two Us and Vs each somehow became fused into a single one. Alternatively, perhaps one copy of each was lost; this might be possible, provided that each sex chromosome carries a dominant factor ensuring the respective sex organ development. However, if degeneration is minor, as predicted, the U and V should each carry many genes, and dosage relationship imbalances should prevent one copy being lost from such polyploid bryophytes (unless the relative dosage of genes carried on the U and V to autosomal genes is unimportant). Polyploids in haploid plant groups are thus interesting for evaluating the idea that, in species with genetically degenerating sex chromosomes, dosage relationships lead to the evolution of dosage compensation (Pessia et al. 2012).

Most of the autosomes were found as homeologous pairs, as expected after a WGD event. However, only 12 autosomes were found (not 14, the expected number after the polyploidisation event). Based on regions carrying the same genes in the same order, the studies identified 5 autosome pairs in both sequenced mosses, but two autosomes were found only once. Their missing homeologues appear to have become parts of the sex chromosomes, explaining the observed autosome number, and also explaining the high sex chromosome gene number mentioned above (about double the roughly 7% of the species’ genes that might be expected with a haploid number of 13).

Since the U and V assembly sizes are both similarly large, they presumably each gained both of these two former autosomes, which were probably added to the recombining (pseudo-autosomal region, or PAR) end of an ancestral U or V chromosome, and then recombined onto the other. Such a process probably created the ‘X-added region’ of Eutherian mammal sex chromosomes (Skaletsky et al. 2003, Waters et al. 2001). Addition to both members of a sex chromosome pair might be favoured as it ensures reliable segregation of the ancestrally autosomal element (Blackmon and Demuth 2015). In the few cases of sex chromosome-autosome fusions so far studied in other bryophytes, this appears not to have happened (Renner et al. 2017); in these liverworts, perhaps the fusions added the autosomes onto non-recombining regions of the U or V chromosomes, so that addition to the other sex chromosome would have required a second fusion.

If the neo-sex chromosome resulting from fusions in the moss ancestor initially had a greatly expanded recombining region carrying the former autosome’s genes. It is therefore interesting to ask whether any PAR is still present. Genetic mapping identified PARs at both ends of the *C. purpureus* U and V chromosomes (McDaniel et al. 2007), based on a small number of AFLP markers (which may not imply a physically small size, as repetitive sequences in the UV-linked region could provide disproportionately many such markers per megabase). However, the high repetitive content and rearranged order of sequences on the U and V suggest that all the added (formerly recombining) regions have stopped recombining and changed from their autosomal states, again resembling the evolutionary history of the Eutherian mammal Y chromosome.

In mammals, several distinct recombination suppression events produced sets of Y-linked alleles with successively smaller divergence from their X-linked alleles, named ‘evolutionary strata’ (Lahn and Page 1999, Skaletsky et al. 2003), indicating clearly that the loss of recombination was not a direct consequence of the addition event. In *C. purpureus*, each of the two added autosomes must have lost recombination separately. However, unlike the mammalian XY PARs, the present moss chromosome assemblies detected no terminal region with autosomal-like low repetitive sequence content, or any region in which the U and V are collinear (non-rearranged). Information from the genome sequences could help identify genetic markers, including microsatellites, that can be used to test genetically whether PARs do exist, and estimate their sizes—an example where sequencing does not answer a major question about a genome, but classical genetics can help.

As outlined above, gene losses are predicted to be minor in haploid plants, yet many genes were found on the *C. purpureus* U and V (more than 3000 transcripts each). Although the authors conclude that lack of recombination has not caused degeneration in this moss, it is difficult to distinguish whether these large gene numbers definitively indicate the predicted lack of degeneration, or simply reflect the autosomal additions, the latest of which could have been too recent for much degeneration to occur. It is not known how many genes were originally present on the autosomes that were added to the U or V, but a rough estimate is possible, from their extant homeologues. Together, the autosomes constitute about 69% of the total assembly length. If the total gene number in *C. purpureus* is similar to the estimated 16,545 protein-coding genes in *S. caninervis* (Silva et al. 2021), its autosomes may thus carry around 13,100 genes in total; since autosomes 2 and 9 represent 18% of the genome, around 2500 genes might have been added to the U and V (assuming similar gene densities for all autosomes, and no differential re-diploidisation after the WGD). This agrees fairly well with the numbers inferred in the assembly, so it appears consistent with the prediction of few losses from either the U or the V. Losses are, however, discussed further below.

A large proportion of the many Dicranidae species whose chromosome numbers have been studied have chromosome numbers exceeding the ancestral number of 8 (Rice et al. 2014), consistent with a WGD event before their radiation. This is supported by high synonymous site divergence (Ks), exceeding 60%, between paralogous genes in *C. purpureus* inferred to have been created in the WGD (for synonymous sites, selective constraints should not be strong, and such high divergence indicates a long evolutionary time). Ks for 338 extant *C. purpureus* U–V gene pairs suggests that they stopped recombining much more recently, averaging only 2–3%, with most estimates below 10%. Therefore, either the fusions with autosomes that created the present U–V gene pairs both occurred long after the WGD, or else recombination in both added regions continued for a long time afterwards (but stopped long enough ago for considerable accumulation of repetitive sequences, and for gene orders to become rearranged between the U and V). It will be interesting to estimate divergence between genes on the U and V chromosomes and their paralogues on each of the respective autosomal progenitors. Comparisons with the progenitor autosomes’ gene contents should also yield estimates of the extent of degeneration in the times since recombination was suppressed.

Other Dicranidae species should also be studied. Chromosome numbers differ among these species (Rice et al. 2014), suggesting the possibility of independent fusions during the radiation (rather than one event before their radiation). These might involve various autosomes, and perhaps sometimes only one sex chromosome. The possibility of placing such events in their order of occurrence using sequence divergence values, and identifying the sex-linked genes in different species, may make these mosses excellent for studying how fusions to sex chromosomes affect their subsequent evolution, including the gene movements mentioned below.

In *C. purpureus*, gene tree clustering identified 101 genes present only on the U chromosome, and 209 only on the V, as well as 338 U–V single-copy gene pairs present on both (62 further genes were present on both, but not single-copy on both). All 648 single-copy genes were presumably once present on both the U and V, but only 53% are still present on both, while around 85% have now been lost from the U and 68% from the V. These numbers support the prediction that degeneration should affect both haploid sex chromosomes roughly equally, but not that gene losses should be minor. It will be important to disentangle the contributions from gene losses from the most recently added autosome, versus other processes. Net losses of U- and V-linked genes also depend on duplications of autosomal genes onto the sex chromosomes (Hughes 2015), and possible gene movements in the opposite direction (Albritton et al. 2014, Sturgill et al. 2007).

The loss of genes after a neo-sex chromosome stopped recombining might be particularly rapid in a polyploid species such as this moss; as noted above, two formerly autosomal regions both appear to have stopped recombining, and their gene functions might have been preferentially maintained by their homeologous autosomal copies. It will therefore be interesting to compare losses from the member that remained as an autosome versus losses from the one that became sex linked. The latter should preferentially begin losing genes, while diploidisation of the other chromosome should slow down after its homeologue became sex linked.

As Table 1 shows, the only genes expected to be lost from haploid sex chromosomes are ones with specific expression patterns: genes expressed specifically in one sex of gametophyte, can be lost from the U chromosome or the V, as can sporophyte-specific genes if hemizygosity does not greatly reduce fitness. It will therefore be very interesting to analyse genes losses in relation to their expression patterns. The authors conclude that the genes that are still found on both the U or V of *C. purpureus*, are unexpectedly often involved in male or female ‘co-expression modules’. This may reflect specific male or female gametophyte functions, and it will be interesting to test whether genes with expression indicating such functions are indeed preferentially lost, compared with genes with other expression patterns. However, if the presence of functional autosomal paralogous copies allows losses in polyploid species, non-polyploids may offer clearer test*s*.

A final interesting question is whether the sex chromosome of this moss are homologues of those in other bryophytes. This can potentially be studied using the genome sequence of the distant relative, *Marchantia polymorpha* (a liverwort), whose U- and V-linked regions include modest numbers of genes (Marks et al. 2019), which should include the sex-determining genes. The U-linked female-determiner has recently been identified, with a V-linked copy that appears to be essential for male reproduction (Iwasaki et al. 2021). It may now be possible to test whether its homologue is also on the moss U and/or V, even with the vast evolutionary divergence times between these bryophytes, and the genome duplication, chromosome fusions and rearrangements outlined above. Bryophyte species with separate male and female gametophytes can, however, revert to hermaphroditism, and such transitions are common (Renner et al. 2017, Villarreal and Renner 2013). A recent review estimated that only around 57% of all mosses, 68% of liverworts, and 40% of hornworts (the third type of bryophyte) have separate sexes (Perley et al. 2015). It is thus also possible that different bryophyte lineages have evolved separate sexes independently, involving different sex-determining genes. Such “turnovers” have been detected in animal taxa (reviewed by Vicoso (2019)), and in other plant groups. More studies of other bryophytes that have not undergone WGD events, such as some liverworts (Renner et al. 2017), might allow us to learn whether this occurs in haploid plants also, or whether these sex chromosomes are immune to turnovers (and, if so, to understand why).
